# Improved adsorption and degradation performance by S-doping of (001)-TiO_2_

**DOI:** 10.3762/bjnano.10.206

**Published:** 2019-11-01

**Authors:** Xiao-Yu Sun, Xian Zhang, Xiao Sun, Ni-Xian Qian, Min Wang, Yong-Qing Ma

**Affiliations:** 1 Anhui Key Laboratory of Information Materials and Devices, School of Physics and Materials Science, Anhui University, Hefei 230039, China; 2 School of Electronic Engineering, Huainan Normal University, Huainan 232038, China; 3 Institute of Physical Science and Information Technology, Anhui University, Hefei 230039, China

**Keywords:** anatase, chemical state, degradation, photocatalytic properties, S-doping, thermal chemical vapor deposition, titanium dioxide (TiO_2_)

## Abstract

In this work, sulfur-doped (S-doped) TiO_2_ with the (001) face exposed was synthesized by thermal chemical vapor deposition at 180 or 250 °C using S/Ti molar ratios *R*_S/Ti_ of 0, 0.5, 1, 2, 3, 4 and 5. The S-doped samples synthesized at 250 °C exhibit a significantly improved photocatalytic performance. More precisely, S-doping has the following effects on the material: (1) S can adopt different chemical states in the samples. Specifically, it exists in the form of S^2−^ replacing O^2−^ at a ratio of *R*_S/Ti_ = 1 and also in the form of S^6+^ replacing Ti^4+^ at *R*_S/Ti_ ≥ 2. As a result, S-doping causes a lattice distortion, because the ionic radii of S^2−^ and S^6+^ differ from that of the O^2−^ and Ti^4+^ ions. (2) S-doping increases the adsorption coefficient *A_e_* for methylene blue (MB) from 0.9% to 68.5% due to the synergistic effects of the oxygen vacancies, increased number of surface chemical adsorption centers as a result of SO_4_^2−^ adsorption on the TiO_2_ surface and the larger pore size. (3) S-doping increases the MB degradation rate from 6.9 × 10^−2^ min^−1^ to 18.2 × 10^−2^ min^−1^ due to an increase in the amount of •OH and •O^2−^ radicals.

## Introduction

Anatase TiO_2_ with a tetragonal symmetry has widely been used for the degradation of organic pollutants, as well as in electrocatalysis, solar cells and self-cleaning applications. Its wide use is based on its physicochemical properties, abundance, nontoxicity, environment-friendliness and low cost [1–7]. The photocatalytic properties of anatase TiO_2_ crystals are anisotropic since the differently exposed crystal faces have different atomic and electronic structures and surface energy. This results in differences in the adsorption capacity regarding pollutant molecules and in the electron transfer properties of TiO_2_ [8–9]. It is widely believed that the exposed (001) face has a high photocatalytic activity [10]. However, Yu et al. demonstrated that an appropriate proportion of exposed (001) and (101) crystal faces, which forms a “surface heterojunction”, facilitates the separation of photo-generated carriers [8]. Consequently, this improves the photocatalytic performance. Wang et al. reported that TiO_2_ with an ideal (001) face was inert to both methanol and water, and the activity of the (001) face was only enhanced after surface reduction or reoxidation [11].

It is well known that the conduction band of anatase TiO_2_ is composed of the Ti 3d state and the valence band mainly comprises the O 2p states, with a band gap energy (*E_g_*) of 3.2 eV. Therefore, the photo-excitation of electron–hole pairs requires photon energies *hν* ≥ 3.2 eV (wavelength λ < 387 nm). This means that the photo-response range of TiO_2_ lies in the ultraviolet region, and it can only absorb less than 5% of the total energy of the solar spectrum [12]. Thus, increasing the spectral response range of TiO_2_ has become an important research area [13].

Significant efforts have been devoted to the posttreatment of the exposed (001) face of TiO_2_ [denoted by (001)-TiO_2_] to further improve its photocatalytic performance. Li et al. synthesized composites of mesoporous (001)-TiO_2_ and C applying a one-pot hydrothermal strategy in the presence of glucose and hydrofluoric acid (HF). The composites an exhibited excellent visible-light-driven photocatalytic performance [14]. Chen et al. synthesized a composite of g-C_3_N_4_ and B-doped (001)-TiO_2_ via a solvothermal method in order to improve the visible-light photocatalytic activity [15]. Cao et al. used first-principles simulations to study the electronic and optical properties of (001)-TiO_2_ and MoS_2_ composites. Their results suggested that the effective photosensitization of MoS_2_ and the stable interface between the two phases could promote the transfer of electrons from MoS_2_ to (001)-TiO_2_ and enhance its visible-light response [16]. It was also demonstrated that Au nanoparticles deposited on the surface of (001)-TiO_2_ particles could promote the separation of photo-generated carriers, improve cycle stability and enhance the visible light response [17–18].

In addition to the composite two-phase approach described above, several groups reported elemental substitution in (001)-TiO_2_ with the aim of improving its photocatalytic performance. For example, a theoretical study on C/F-codoped (001)-TiO_2_ concluded that C/F atoms preferentially replaced O atoms on the (001) face, resulting in a surface conduction layer that could promote the migration of photo-generated carriers [19]. N/P-codoping of (001)-TiO_2_ resulted in a reduction of the band gap from 3.20 to 2.48 eV [20]. To the best of our knowledge, S-doped (001)-TiO_2_ has not yet been investigated. However, the S-doped non-(001)-TiO_2_ is well reported in literature [12,21–26]. Moreover, in previous reports, S-doping was mainly performed using solid-state calcination or hydrothermal methods. The solid-state calcination results in the aggregation of particles. For S-doping via hydrothermal methods, the precursors were placed in an oven or a muffle furnace and heated to induce the reaction.

Although some progress has already been made concerning S-doped TiO_2_, there are many issues that require further investigation. These include the differences between lightly and heavily doped TiO_2_ as well as the effects of S-doping on the crystal structure, the energy band structure and the chemical states of Ti and O. In this work, (001)-TiO_2_ nanoparticles (NPs) were first prepared, then S-doping was performed by thermal chemical vapor deposition. We observed that S-doping greatly enhances the photocatalytic performance of (001)-TiO_2_, and we revealed the related mechanism by a systematic investigation of the material.

## Experimental

### Synthesis of nanoparticles

20 mL of tetrabutyl titanate (TBT, 99%, Aladdin) was put in a Teflon-lined stainless steel autoclave. Then, 5 mL of deionized water and 5 mL of a HF acid solution (hydrofluoric acid, 40%, Aladdin) were added sequentially. The autoclave was maintained at 180 °C for 8 h and then cooled naturally to room temperature. The obtained precipitate was washed several times with deionized water and absolute ethanol and then dried at 60 °C to obtain the resulting (001)-TiO_2_ NPs.

S-doping of the (001)-TiO_2_ NPs was performed by thermal chemical vapor deposition. First, 300 mg of (001)-TiO_2_ NPs was added to a beaker containing 100 mL deionized water. Then, the desired amount of thiourea (99%, Aladdin) was added; the molar ratio of S in the thiourea to Ti in TiO_2_ (*R*_S/Ti_) was chosen 0, 0.5, 1, 2, 3, 4 and 5. After magnetic stirring for 30 min, the solution was transferred to a 120 mL quartz crucible that was subsequently placed inside a 500 mL Hastelloy autoclave, and 150 mL of deionized water was added to the autoclave. The autoclave was then heated to 180 °C or 250 °C and maintained at this temperature for 12 h. The reaction occurred in the quartz crucible under the environment of high-temperature vapor of deionized water. The thermal chemical vapor deposition has the advantages of rapid heat transfer, uniform heating and acceleration of the diffusion of S atoms into the TiO_2_ crystal lattice. After the autoclave had cooled naturally to room temperature, the precipitate was washed several times with deionized water and absolute ethanol, respectively and then dried at 60 °C. The S-doped TiO_2_ samples synthesized at 180 °C were named 1-S0, 1-S0.5, 1-S1, 1-S2, 1-S3, 1-S4, and 1-S5; samples synthesized at 250 °C were named as 2-S0, 2-S0.5, 2-S1, 2-S2, 2-S3, 2-S4, and 2-S5.

### Characterization

The crystal structure of the samples was investigated using an X-ray diffractometer (XRD, Rigaku Industrial Corporation, Osaka, Japan) with Cu Kα radiation (λ = 1.5406 Å, operated at 40 kV and 100 mA). Transmission electron microscopy (TEM; JEM-2100, JEOL, Tokyo, Japan) was used to characterize the morphology of the samples. Ultraviolet–visible diffuse reflectance spectra (UV–vis DRS) of the samples were measured on a Shimadazu U-4100 spectrometer (U-4100, Shimadazu Corporation, Tokyo, Japan). X-ray photoelectron spectroscopy (XPS) was performed using a Thermo Scientific ESCALAB 250Xi (Thermo Scientific Inc., USA). The chemical bonds of the photocatalysts were probed by Fourier-transform infrared (FTIR) spectroscopy (Vertex 80/Hyperion2000, Bruker, Germany). The Brunauer–Emmett–Teller (BET) specific surface areas were calculated based on the N_2_ adsorption–desorption isotherms measured at 77 K using a gas adsorption apparatus (Autosorb-iQ, Quantachrome Instruments, USA). The pore size distribution was calculated using the Barret–Joyner–Halenda (BJH) method. The photoluminescence (PL) was measured on a fluorescence spectrophotometer (F-4500, Hitachi, Japan). Electron spin resonance (ESR) signals of the reactive species spin trapped by 5,5-dimethyl-1-pyrroline-*N*-oxide (DMPO) were determined on a Bruker EMX plus 10/12 (equipped with Oxford ESR910 Liquid Helium cryostat). For detection of the superoxide radicals (•O_2_^−^) and hydroxyl radicals (•OH), 2.5 mg of the photocatalyst was dispersed in 1 mL DMPO/methyl alcohol solution or DMPO/H_2_O solution.

The photocatalytic activity was tested by the degradation of methylene blue (MB). For this, the samples were placed 20 cm away from a xenon lamp (300 W, 16 A). The experimental process was as follows: 50 mg of catalyst was added into a 100 mL MB solution with a concentration of 10 mg/L, and the samples were kept in a dark room for 30 min to achieve the adsorption–desorption equilibrium. Subsequent to irradiation, samples of the solution were taken every 10 min. After high-speed centrifugation, the concentration of MB was analyzed by a UV–vis spectrometer (UV-3200S, MAPADA, Shanghai, China) and calculated using a calibration curve.

## Results and Discussion

**Figure 1:**
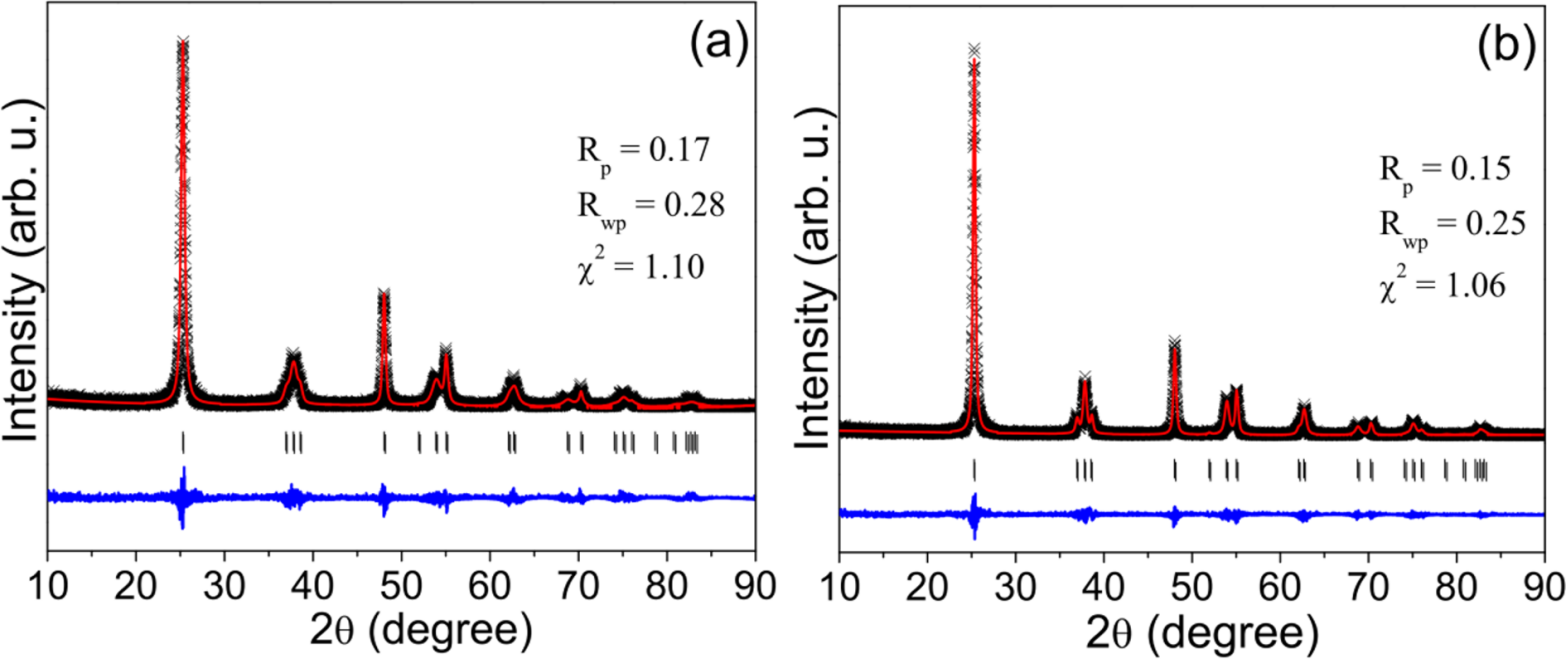
Experimental (×) and calculated (—) X-ray powder diffraction patterns of 1-S0 (a) and 2-S2 (b). Peak positions are shown as small markers (|). The lower trace represents the difference between the calculated and experimental data.

The crystal structures of all samples were characterized by XRD. Figure 1 shows the experimental data and the results calculated by Rietveld refinement of 1-S0 (a) and 2-S2 (b). The calculated results match well with the experimental data indicating that all the TiO_2_ samples have a single-phase anatase structure with tetragonal symmetry and space group *I*4_1_/*amd*. The unit cell parameters of the undoped 1-S0 samples were *a* = 3.7852 Å and *c* = 9.5139 Å. We observed a minimal change for the S-doped samples synthesized at 180 °C; however, we found a drastic change in the samples synthesized at 250 °C, as shown in Table 1. This structural change results from the S atoms entering the lattice. The *c*/*a* ratio gradually increases from 2-S0.5 to 2-S3 and reaches a maximum for the 2-S3 sample before it decreases again for the 2-S4 and 2-S5 samples. The reason for the variation of *c*/*a* with *R*_S/Ti_ will be discussed in detail in section below along with the XPS results.

**Table 1:** The unit cell parameters *a*, *c* and the *c*/*a* value for S-doped (001)-TiO_2_ at 250 °C.

	2-S0	2-S0.5	2-S1	2-S2	2-S3	2-S4	2-S5

*a* (Å)	3.7852	3.7876	3.7867	3.7866	3.7860	3.7864	3.7859
*c* (Å)	9.5139	9.5120	9.5091	9.5131	9.5131	9.5069	9.5083
*c*/*a*	2.5135	2.5114	2.5118	2.5125	2.5127	2.5108	2.5115

**Figure 2:**
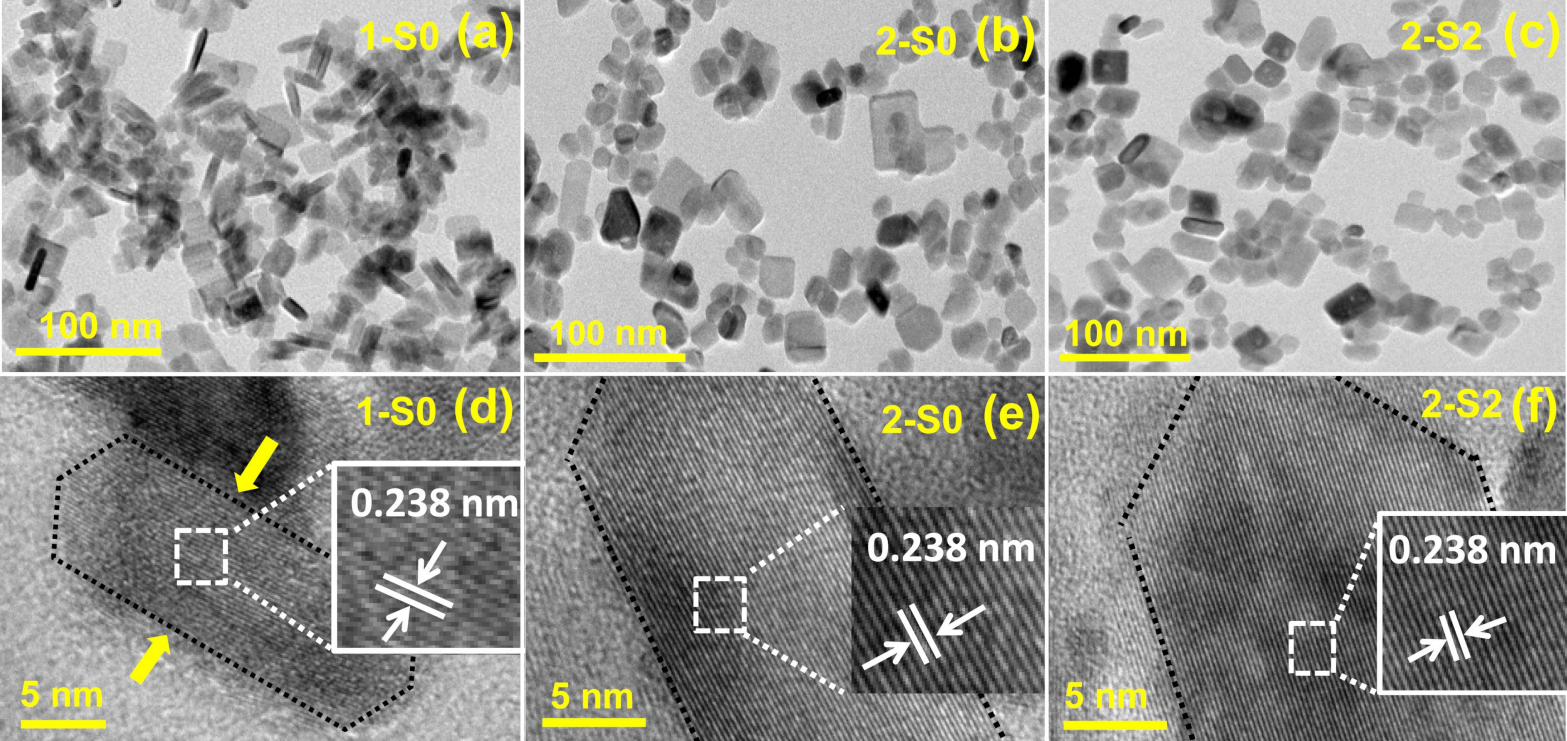
TEM images (a–c) and HRTEM images (d–f) for 1-S0, 2-S0, and 2-S2.

Figure 2 shows the TEM and HRTEM images of the 1-S0 (a, d), 2-S0 (b, e), and 2-S2 (c, f) samples. Obviously, the undoped 1-S0 sample synthesized at 180 °C is composed of square sheet-like particles, which is the typical morphology of (001)-TiO_2_ [8,27–28]. The HRTEM image (Figure 2d) of the particle side shows a lattice fringe spacing of 0.238 nm. This corresponds to the (004) crystal face of TiO_2_ and indicates that the top and bottom square surfaces (indicated by the arrow) are the (001) faces [29]. For the samples synthesized at 250 °C, the TEM images (Figure 2b and Figure 2c) of the undoped 2-S0 and S-doped 2-S2 show that the edges and corners of some of the square particles become blurred. The HRTEM images (Figure 2e and Figure 2f) of the particles also exhibit lattice fringes associated with the (004) crystal face.

**Figure 3:**
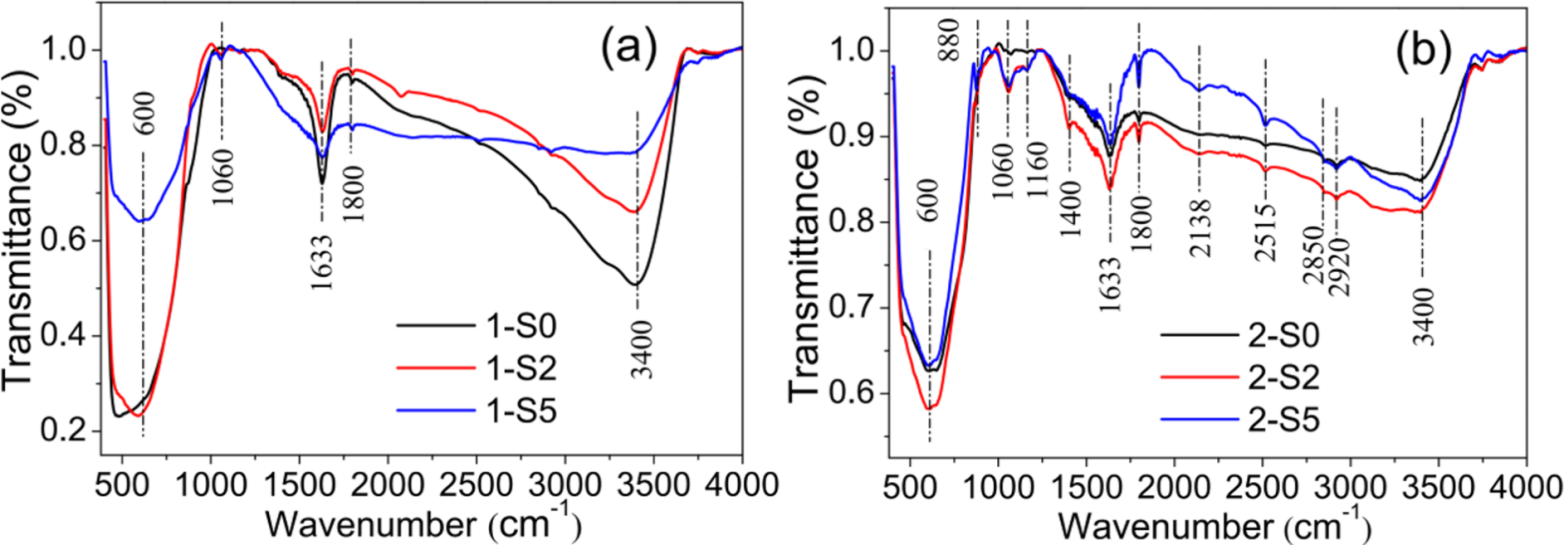
The FTIR spectra of 1-S0, 1-S2 and 1-S5 (a); 2-S0, 2-S2 and 2-S5 (b).

FTIR spectra were measured for all the samples. Figure 3 shows the results of the samples with *R*_S/Ti_ = 0, 2 and 5. The positions of the absorption peaks and the corresponding assignments to vibrational modes are listed in Table 2. In contrast to the undoped 1-S0 and 2-S0 samples, the S-doped samples exhibit the Ti–S vibration mode with the corresponding absorption peak located at 1060 cm^−1^. Compared to the spectrum of the S-doped samples synthesized at 180 °C (Figure 3a), the spectrum of the S-doped samples produced at 250 °C (Figure 3b) exhibits the following differences: (1) the Ti–S vibration is stronger, (2) a new vibrational mode appears, i.e., the Ti–O–S vibration at 1160 cm^−1^ [21] caused by S^6+^ replacing Ti^4+^ (this was also confirmed by the XPS results given below), (3) the S=O vibration in the range of 1380–1400 cm^−1^ results from a sulfate complex formed by surface-adsorbed SO_4_^2−^ and TiO_2_ [30–31], (4) the vibrational modes at 1800 and 2515 cm^−1^ can be attributed to the –COOH group [32–33]; while those at 2850 and 2920 cm^−1^ can be attributed to the C–H group [34]; the C–OH vibration mode is located at 880 cm^−1^ [35–36], (5) the vibrational mode at 2138 cm^−1^ is assigned to the stretching vibration of the C–N group, which is a residue of the thiourea decomposition [37–38].

**Table 2:** Position of the FTIR absorption peaks and the corresponding vibrational modes.

position (cm^−1^)	vibrational mode	references

425–840	Ti–O or Ti–O–Ti	[37–38]
880	alkoxy C–OH stretching mode	[32–33]
1060	Ti–S	[28]
1160	Ti–O–S	[19]
1400	S=O stretching mode	[29]
1800, 2515	C=O and O–H stretching modes of –COOH in organic residues	[30–31]
1633, 3400	flexural vibrations of O–H in free water molecules	[39–40]
2850, 2920	C–H stretching mode	[34]
2138	C–N stretching mode	[35–36]

In order to investigate the variation of the chemical states (CSs) of the S-doped (001)-TiO_2_ as a function of the *R*_S/Ti_, core level XPS of the Ti 2p, O 1s and S 2p regions was performed for the samples 1-S0, 1-S2 and 1-S5. The results are not shown here because the S element was not detected possibly be due to the small amount of S atoms doped into the samples or adsorbed at the TiO_2_ surface [39]. By fitting the XP spectra of Ti 2p and O 1s we deduce that the CSs correspond to TiO_2_ and TiO*_x_* for Ti [40–41] and TiO_2_ and –OH for O [42–43]. Moreover, the ratios of the CSs of Ti and O in TiO_2_ do not change significantly with *R*_S/Ti_ when S-doping is carried out at 180 °C.

**Figure 4:**
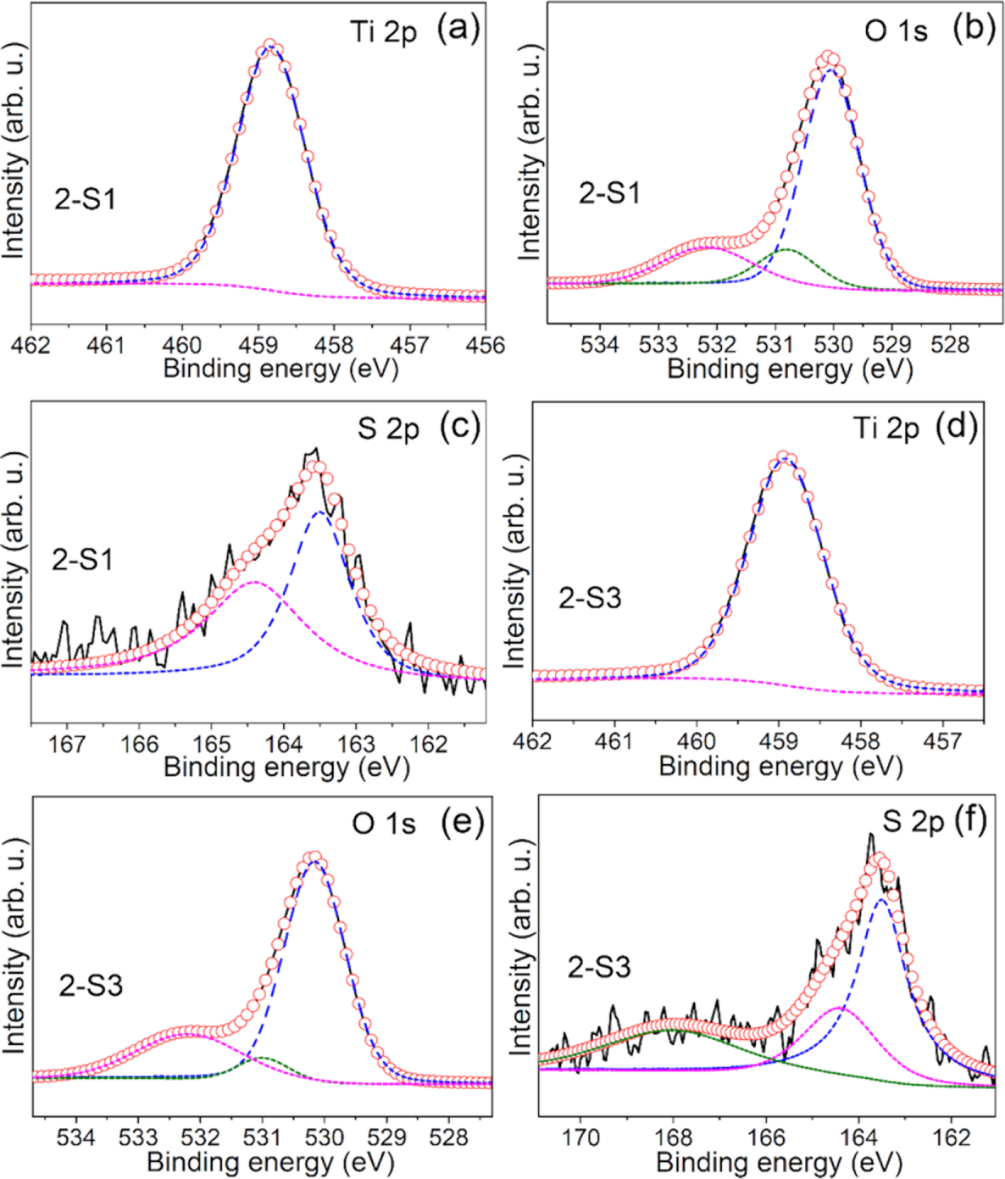
Core-level XP spectra of Ti 2p (a and d), O 1s (b and e) and S 2p (c and f) for 2-S1 and 2-S3. The solid line is the experimental curve, the dashed line is the fitted curve, and the open circles are the sum of the fitted curves.

The core-level XP spectra of the Ti 2p, O 1s and S 2p regions for all the S-doped samples synthesized at 250 °C were measured, and Figure 4 representatively shows the results for 2-S1 and 2-S3. The chemical states of Ti, O and S, the corresponding binding energies (BE) and the CS ratios derived for 2-S0, 2-S0.5, 2-S1, 2-S2, 2-S3, 2-S4 and 2-S5 are listed in Table 3.

**Table 3:** The chemical states (CSs) of Ti, O and S and the corresponding binding energies (BE) and CS ratios derived for 2-S0, 2-S0.5, 2-S1, 2-S2, 2-S3, 2-S4, and 2-S5.

250 °C		Ti			O		S		
	CS	TiO_2_	TiO_x_	TiO_2_	–OH	O_ν_	Ti–S	S	S^6+^

2-S0	BE (eV)	458.8	460.2	530.0	531.0	532.7	–	–	–
	ratio (%)	98.5	1.5	80.6	15.1	4.3	–	–	–
2-S0.5	BE (eV)	458.9	460.2	530.1	530.9	532.4	–	–	–
	ratio (%)	97.2	2.8	74.5	15.6	9.9	–	–	–
2-S1	BE (eV)	458.8	460.2	530.0	530.8	532.2	163.5	164.4	–
	ratio (%)	96.6	3.4	69.7	12.8	17.5	49.5	50.5	–
2-S2	BE (eV)	458.9	460.2	530.1	530.8	532.4	163.5	164.4	168.0
	ratio (%)	97.6	2.4	61.7	19.4	18.9	38.7	22.8	38.5
2-S3	BE (eV)	458.9	460.2	530.2	531.0	532.2	163.5	164.4	168.0
	ratio (%)	96.9	3.1	71.7	5.4	22.9	50.8	23.5	25.7
2-S4	BE (eV)	458.9	460.2	530.1	530.9	532.3	163.5	164.4	168.0
	ratio (%)	97.6	2.4	68.7	9.7	21.6	42.6	35.0	22.4
2-S5	BE (eV)	458.8	460.2	530.0	530.9	532.3	163.5	164.4	168.0
	ratio (%)	97.7	2.3	68.5	11.6	19.9	48.4	37.3	14.3

For all samples synthesized at 250 °C, the XP spectrum of O 1s can be fitted by three peaks and the CSs correspond to TiO_2_, –OH and oxygen vacancies (O_v_) [44]. As the *R*_S/Ti_ increases, the ratio of O_v_ increases from 4.3% (2-S0) to 22.9% (2-S3) and then decreases again to 19.9% (2-S5) (Table 3). The XPS signals of the oxygen vacancies were not detected in the S-doped samples at 180 °C, but they appear in the S-doped samples at 250 °C due to the reducibility of thiourea [45] and the influence of the S-doped element.

Furthermore, the XP spectrum of the S 2p region can be fitted by three peaks, and the CSs correspond to Ti–S, S^0^ and S^6+^ [21,46]. The substitution of O^2−^ by S^2−^ in the TiO_2_ lattice is responsible for the formation of the Ti–S bond [21]. The occurrence of neutral S (S^0^) results from the hydrolysis of thiourea at high temperature [47]. Neutral S (S^0^) is believed to adsorb on the TiO_2_ surface or partially enter the interstitial sites of the TiO_2_ lattice. Some of the S^6+^ ions replace the Ti^4+^ ions in the TiO_2_ crystal lattice, while others exist in SO_4_^2−^ groups [48].

As seen in Table 3, the XPS signal of the S element cannot be detected in 2-S0 and 2-S0.5. For the 2-S1 sample, S replaces O to form the Ti–S bond, which is accompanied by the appearance of S^0^. For the samples with *R*_S/Ti_ ≥ 2, in addition to the chemical states of Ti–S and S^0^, S^6+^ appears which replaces Ti^4+^. For the samples with *R*_S/Ti_ > 2, as *R*_S/Ti_ increases, the proportion of S^6+^ replacing the Ti^4+^ decreases again, while the sum of S^2−^ and S^0^ increases. The ionic radii of S^6+^ and S^2−^ are 0.029 nm and 0.17 nm, respectively, while the ionic radii of Ti^4+^ and O^2−^ in the TiO_2_ lattice are 0.064 nm and 0.122 nm, respectively. As a result, the substitution of Ti^4+^ by S^6+^ or the substitution of O^2−^ by S^2−^ consequently induce a distortion of the crystal lattice [39]. The lattice distortion degree (Δ*R*) is calculated by Δ*R* = *r*_S6+_ (*R*_Ti4+_ − *R*_S6+_) + *r*_S2−_ (*R*_S2−_ − *R*_O2−_), where *r* is the ratio of the CSs and *R* is the ionic radius of the corresponding ion. We calculate Δ*R* values of 0.024 nm (2-S1), 0.032 nm (2-S2), 0.034 nm (2-S3), 0.028 nm (2-S4) and 0.028 nm (2-S5). The 2-S3 sample has the largest Δ*R*, which is consistent with the XRD results.

**Figure 5:**
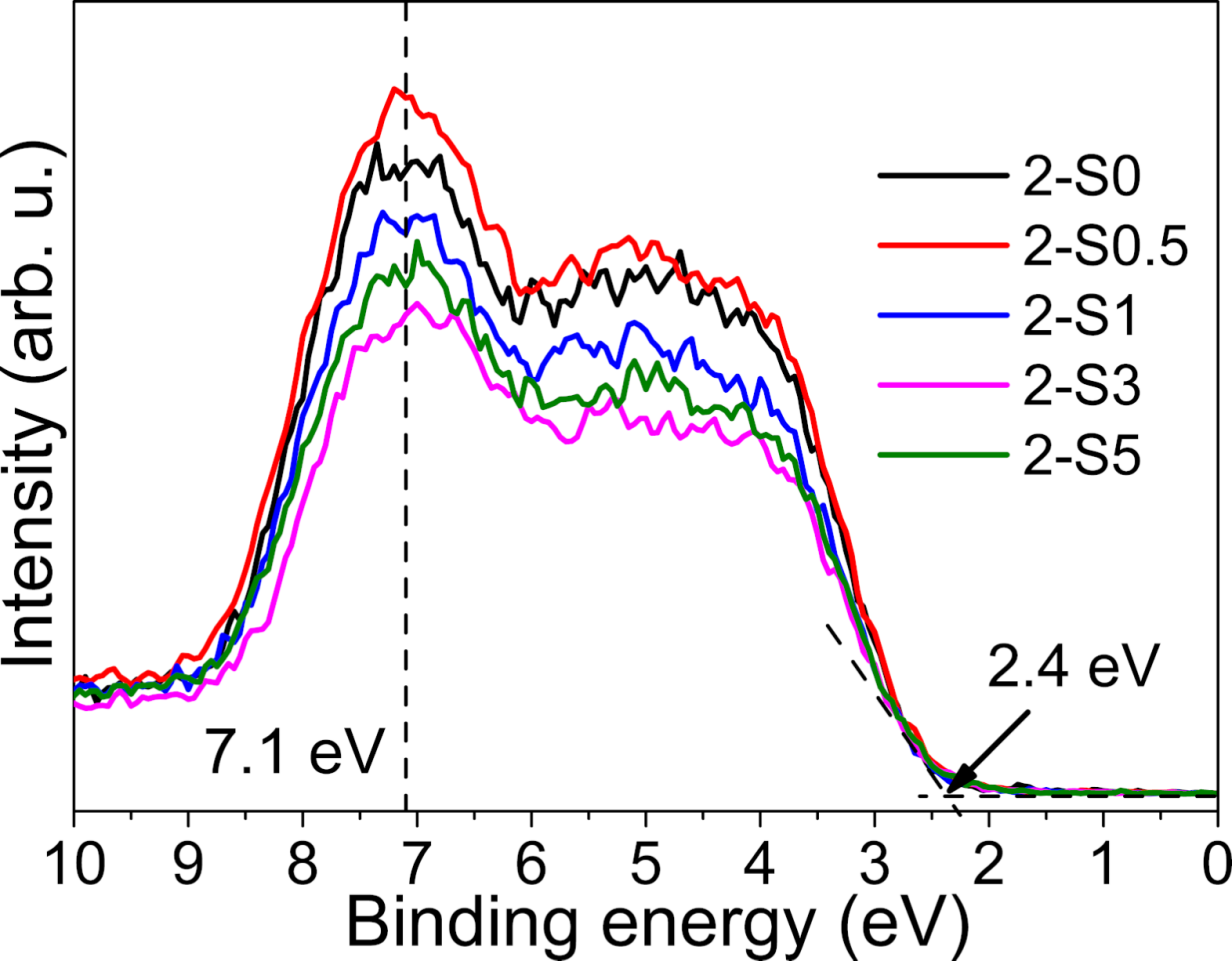
Valence-band XPS of 2-S0, 2-S0.5, 2-S1, 2-S3 and 2-S5.

The XP spectra do not only provide information on the binding energy of the atoms but also on the total density of states (DOS) in the valence band of TiO_2_ [12,49]. In order to investigate if S-doping produces energy levels above the valence band maximum, we measured the valence-band XP spectra of the 2-S0, 2-S0.5, 2-S1, 2-S3 and 2-S5 samples as shown in Figure 5. For all the samples, the valence band maximum is located around 2.4 eV, so S-doping does not shift the valence band maximum towards the forbidden band. Mid-gap states or diffusive states observed in C, N and S-doped TiO_2_ caused by impurities [12] were not observed in our samples. A peak around 7.1 eV is observed in the valence band DOS curve and its intensity decreases in the order of 2-S0.5 > 2-S1 > 2-S5 > 2-S3. The states in the valence band are derived from the O 2p orbitals. At the same time, the ratio of the O_v_ increases in the order of 2-S0.5 < 2-S1 < 2-S5 < 2-S3, as given in Table 3; therefore, the decrease of the DOS mainly results from an increase of the ratio of oxygen vacancies.

**Figure 6:**
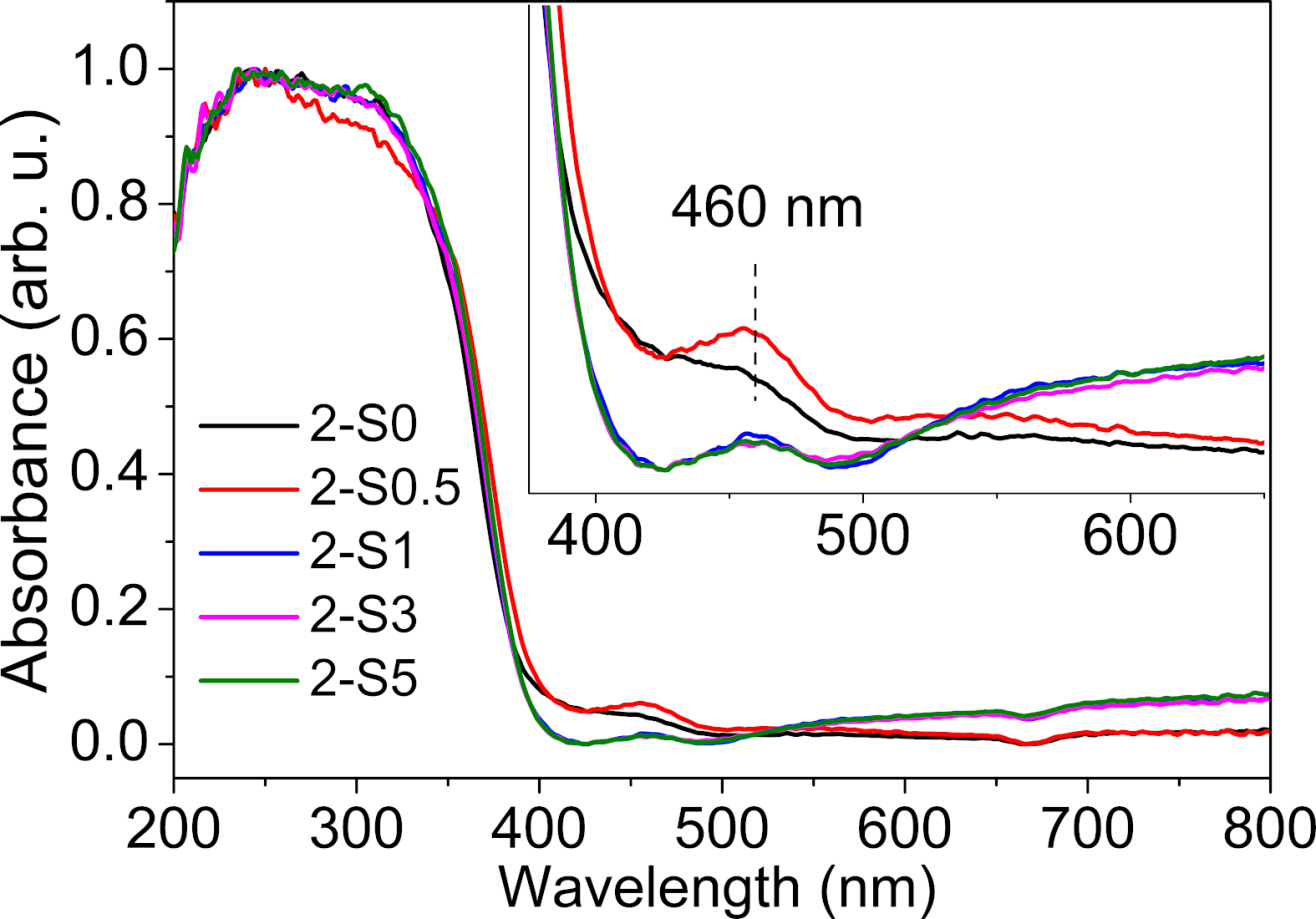
The UV–vis DRS of 2-S0, 2-S0.5, 2-S1, 2-S3 and 2-S5. The inset is the magnified plot of the UV–vis DRS in the visible light region.

Figure 6 shows the UV–vis DRS of 2-S0, 2-S0.5, 2-S1, 2-S3 and 2-S5. The steep absorption edge near 380 nm originates from the intrinsic absorption of TiO_2_, i.e., the absorption results from the transition of electrons from the valence band to the conduction band. The undoped 2-S0 sample exhibits a "tail-like" absorption between 400 and 500 nm, possibly due to surface defects such as oxygen vacancies. The absorption spectrum of the 2-S0.5 sample shows a peak at 460 nm. The visible-light absorption of the samples with *R*_S/Ti_ ≥ 1, where S^2−^ replaces O^2−^ and S^6+^ replaces Ti^4+^, is caused by cooperative effects of the oxygen vacancies and the substituted S elements.

**Figure 7:**
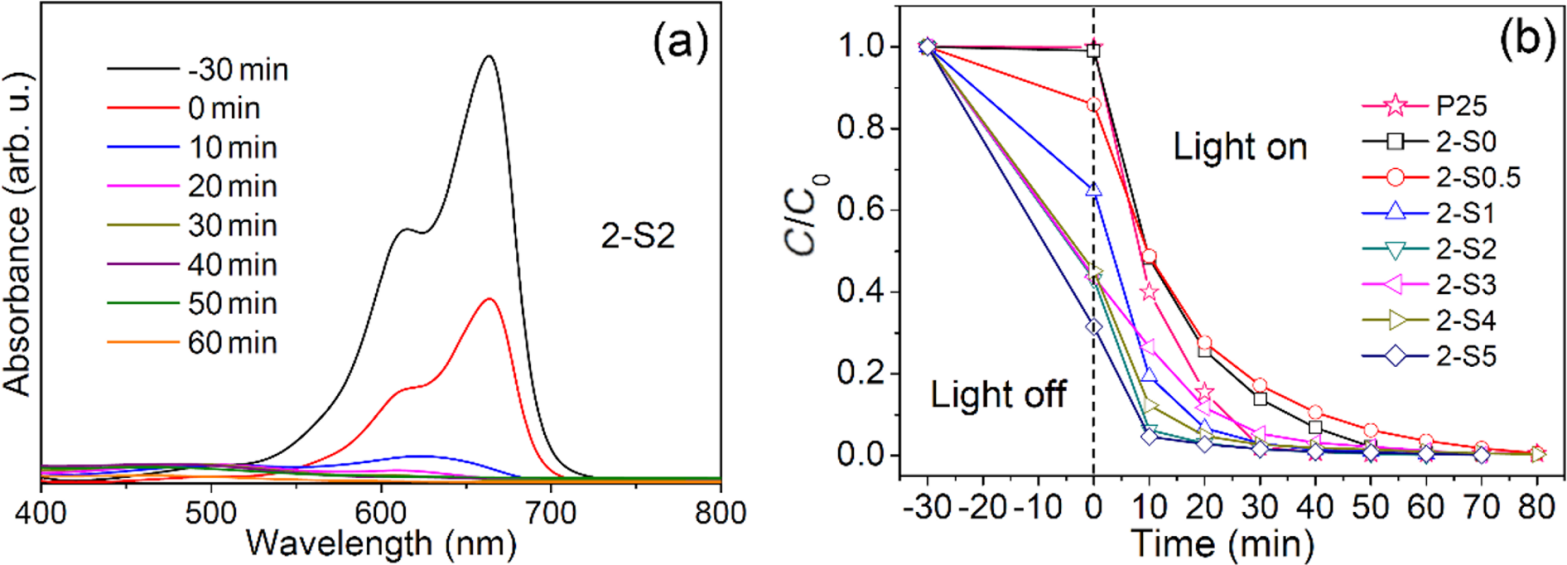
The temporal evolution of the UV–Vis spectra during the photodegradation of aqueous MB over the sample 2-S2 (a) and the variation of the MB concentration *C*/*C*_0_ with time in the presence of 2-S0, 2-S0.5, 2-S1, 2-S2, 2-S3, 2-S4 and 2-S5 and commercial P25 TiO_2_ irradiated by a xenon lamp (b).

The photocatalytic degradation performance of all samples was tested with MB as the target pollutant. Figure 7 representatively shows the temporal evolution of the UV–vis spectra during the photodegradation of aqueous MB over the 2-S2 sample (a) and the variation of the MB concentration *C*/*C*_0_ with time in the presence of 2-S0, 2-S0.5, 2-S1, 2-S2, 2-S3, 2-S4 and 2-S5 irradiated by a xenon lamp (b). For comparison, the performance of commercial P25 TiO_2_ was also tested by the same method as shown in Figure 7b. The negative time scale denotes the adsorption process of MB in the dark, and the positive time scale denotes the photodegradation process of MB during irradiation. The concentration of MB at *t* = −30 min is referred to as the initial concentration *C*_0_. After 30 min of adsorption, the concentration decreases to *C_e_* at *t* = 0. The adsorption efficiency (*A_e_*) is calculated by (*C*_0_ − *C_e_*)/*C*_0_. The degradation efficiency (*D_e_*) is calculated by (*C_e_* − *C*)/*C_e_*, where *C* is the concentration at an irradiation time *t*. The degradation process can be fitted using a pseudo first-order kinetic model ln[*C_e_*/*C*] = *K_app_·t*, where *K_app_* is the apparent reaction rate constant. The *A_e_*, *D_e_* and *K_app_* values calculated for all samples are listed in Table 4 and Table 5.

**Table 4:** The values of the adsorption efficiency (*A_e_*), degradation efficiency (*D_e_*) and the apparent reaction rate constant *K_app_* for the samples prepared at 180 °C.

sample	1-S0	1-S0.5	1-S1	1-S2	1-S3	1-S4	1-S5

*K_app_* (10^−2^ min^−1^)	1.7	1.5	1.8	1.9	3.5	3.7	3.5
*A_e_* (%)	1.9	0	0	0	0.7	5.7	1.0
*D_e_* (%) (*t* = 120 min)	98.4	92.5	95.6	97.8	99.8	99.8	99.8

**Table 5:** The values of the adsorption efficiency (*A_e_*), degradation efficiency (*D_e_*), and the apparent reaction rate constant *K_app_* for the samples prepared at 250 °C and for P25 TiO_2_.

sample	P25	2-S0	2-S0.5	2-S1	2-S2	2-S3	2-S4	2-S5

*K_app_* (10^−2^ min^−1^)	7.5	6.9	5.5	11.7	18.2	6.2	12.1	17.6
*A_e_* (%)	0.08	0.9	14.1	35.1	56.9	56.3	54.8	68.5
*D_e_* (%) (*t* = 120 min)	99.5	99.8	99.4	99.5	99.8	99.6	99.4	99.7

As obvious from Table 4, the samples 1-S0 to 1-S5 hardly adsorb any MB, and the corresponding adsorption coefficients range from 0 to 5.7%. However, the adsorption coefficients of samples 2-S0.5 to 2-S5 are significantly enhanced ranging from 0.9 to 68.5%. Generally, the larger the specific surface area of the sample, the stronger the adsorption capacity. However, the calculated BET surface areas (*S*_BET_), given in Table 6, show that the specific surface areas of samples 2-S0.5 to 2-S5 are smaller than those of samples 1-S0 to 1-S5. Microscopically, the adsorption capacity of TiO_2_ to water or organic pollutant molecules is determined by the amount of and the space between oxygen vacancies (or Ti^3+^) on the surface of the TiO_2_ particles [50]. The adsorption coefficient (*A_e_*) and the ratio of oxygen vacancies almost uniformly change with *R*_S/Ti_. This indicates that the adsorption is mainly determined by the oxygen vacancies. Additionally, the SO_4_^2−^ ions adsorbed on the surface of the S-doped samples synthesized at 250 °C produce acidic sites on the TiO_2_ surface [31,51]. These acidic sites provide more chemical adsorption centers for reactants and oxygen molecules, thus, enhancing the adsorption effect.

The S-doped samples prepared at 180 °C are able to degrade MB within 120 min, and the degradation coefficients vary from 92.5% to 99.8%. The S-doped samples synthesized at 250 °C completely degrade the same amount of MB already within almost 80 min, and the degradation coefficients (*D*_e_) exceed 99.4%.

For 1-S0, the undoped sample synthesized at 180 °C, we calculate a *K_app_* of 1.7 × 10^−2^ min^−1^ (Table 4); by S-doping, the *K_app_* values slightly increase, in detail, the *K_app_* value of the 1-S4 sample is the largest with 3.67 × 10^−2^ min^−1^, which is 2.16 times higher than that of 1-S0. For 2-S0, the undoped sample synthesized at 250 °C, a *K_app_* value of 6.94 × 10^−2^ min^−1^ is computed; S-doping significantly increases the *K_app_* values, whereas 2-S2 has the largest *K_app_* value of 18.2 × 10^−2^ min^−1^, which is 2.62 times higher than that of 2-S0 and the commercial P25 TiO_2_.

**Figure 8:**
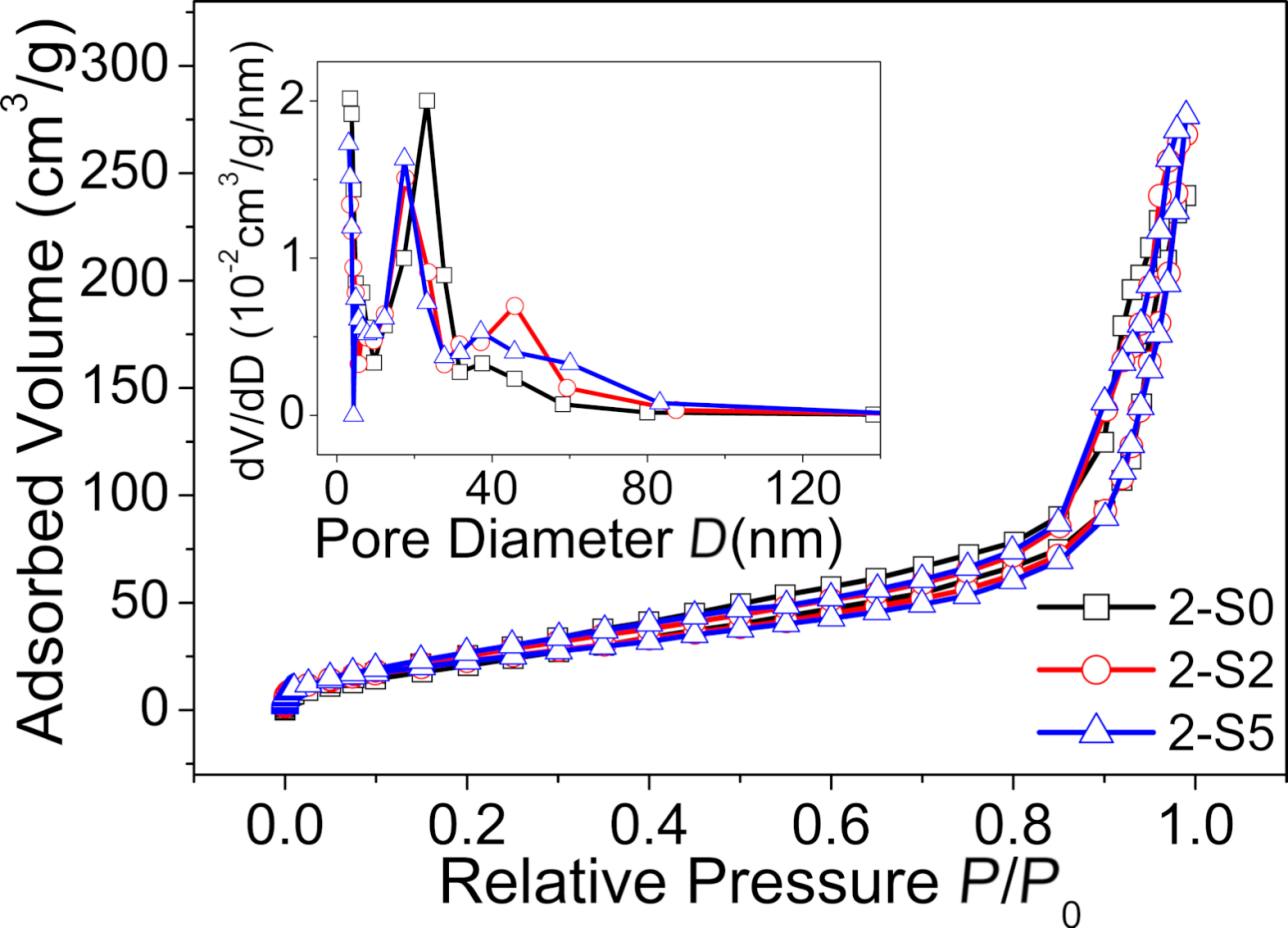
Nitrogen adsorption–desorption isotherms of samples 2-S0, 2-S and 2-S5. The inset shows the pore size distribution calculated using the BJH method.

The *S*_BET_ values of all samples were measured using nitrogen adsorption–desorption isotherms. Figure 8 only shows the results obtained for 2-S0, 2-S2 and 2-S5. The BJH desorption pore distribution (*D_p_*) analysis results are shown in the inset. The *S*_BET_ values and *D_p_* values of all samples are listed in Table 6. Compared to the S-doped samples at synthesized 180 °C, the *S*_BET_ of almost all the S-doped samples prepared at 250 °C are reduced, and with values of 81–123 m^2^g^−1^ they are close to the values reported in literature [52]. This is mainly ascribed to the agglomeration of the TiO_2_ particles synthesized at 250 °C, as shown by the TEM images (Figure 2). In addition, the S-doped samples prepared at 250 °C have a larger pore size, which is beneficial for adsorption of the pollutant molecules.

**Table 6:** Textural parameters of all samples.

sample	1-S0	1-S0.5	1-S1	1-S2	1-S3	1-S4	1-S5

*S*_BET_ (m^2^g^−1^)	174.8	128.8	210.9	128.2	98.4	109.8	145.9
*D_p_* (nm)	8.29	13.9	9.14	11.9	14.5	14.4	11.6
sample	2-S0	2-S0.5	2-S1	2-S2	2-S3	2-S4	2-S5
*S*_BET_ (m^2^g^−1^)	98.6	123.6	81.7	92.1	120.8	89.9	89.6
*D_p_* (nm)	15.0	13.6	18.4	18.0	14.8	17.8	19.1

**Figure 9:**
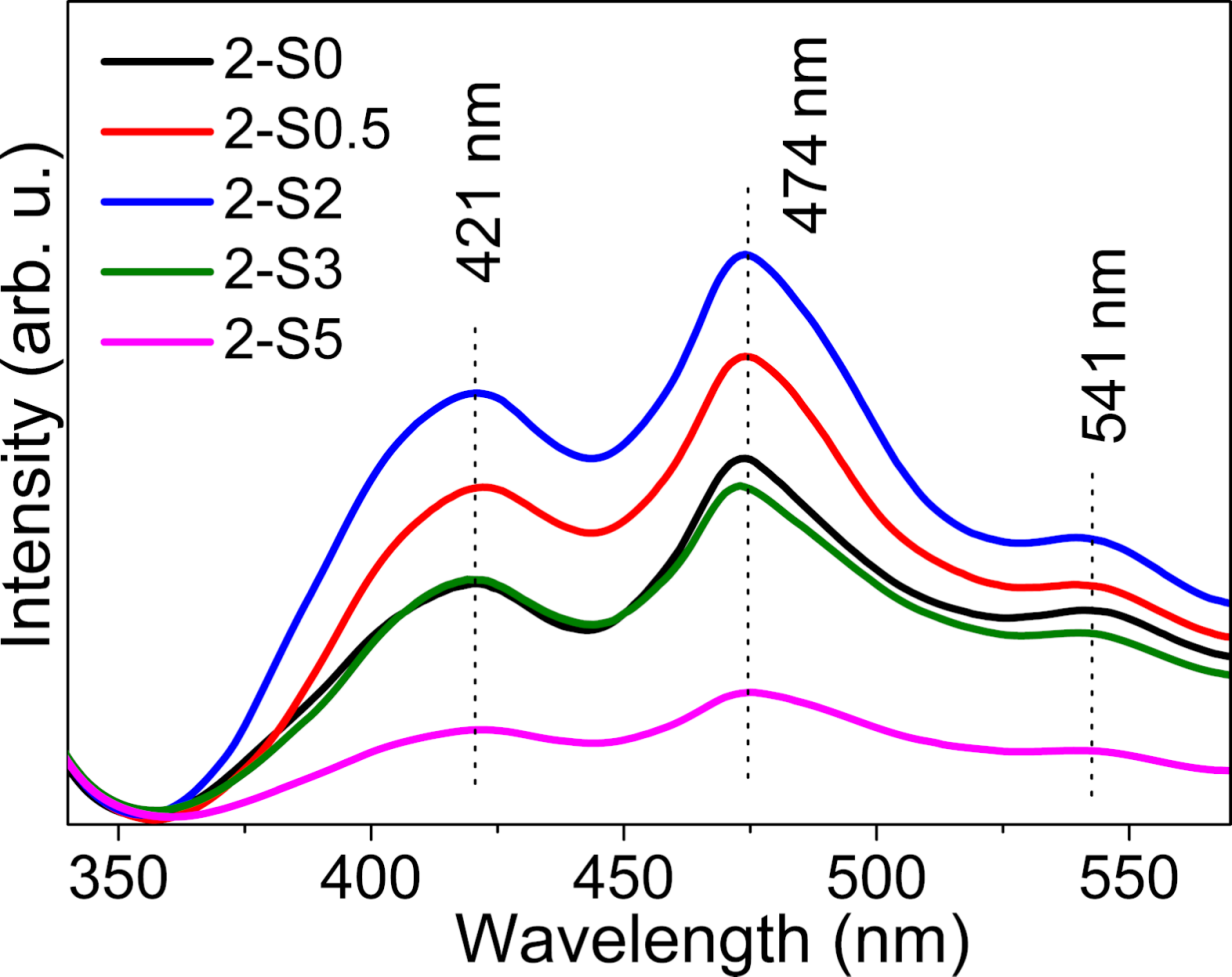
The PL spectra of 2-S0, 2-S0.5, 2-S2, 2-S3 and 2-S5 using an excitation wavelength of λ_ex_ = 300 nm.

PL measurements are effective to examine the separation efficiency and recombination processes of photo-generated carriers, because increased photo-generated electron–hole pair recombination results in a stronger luminescence intensity. Figure 9 representatively shows the PL spectra of the 2-S0, 2-S0.5, 2-S2, 2-S3 and 2-S5 samples. For all the samples, the emission peaks are located at 421, 474 and 541 nm. The emission peak at 421 nm results from the interband transition of TiO_2_. The emission peaks at 474 and 541 nm can be attributed to the radiative recombination of the self-trapped excitons and the hydroxylated Ti^3+^ surface complexes, respectively [53–54]. Obviously, the luminescence intensity initially increases for larger *R*_S/Ti_ with sample 2-S2 having the strongest luminous intensity. Then, for samples 2-S3 to 2-S5, the luminescence intensity decreases again with sample 2-S5 having the weakest intensity. In contrast, no such obvious change of the luminescence intensity with increasing *R*_S/Ti_ has been observed for the S-doped samples at 180 °C. The XPS results indicate that there are various impurities and defects in the S-doped TiO_2_, such as O_v_ (Ti^3+^), –OH, and S impurities; finally, the change of their proportion is most likely the reason for the change in the luminescence intensity.

**Figure 10:**
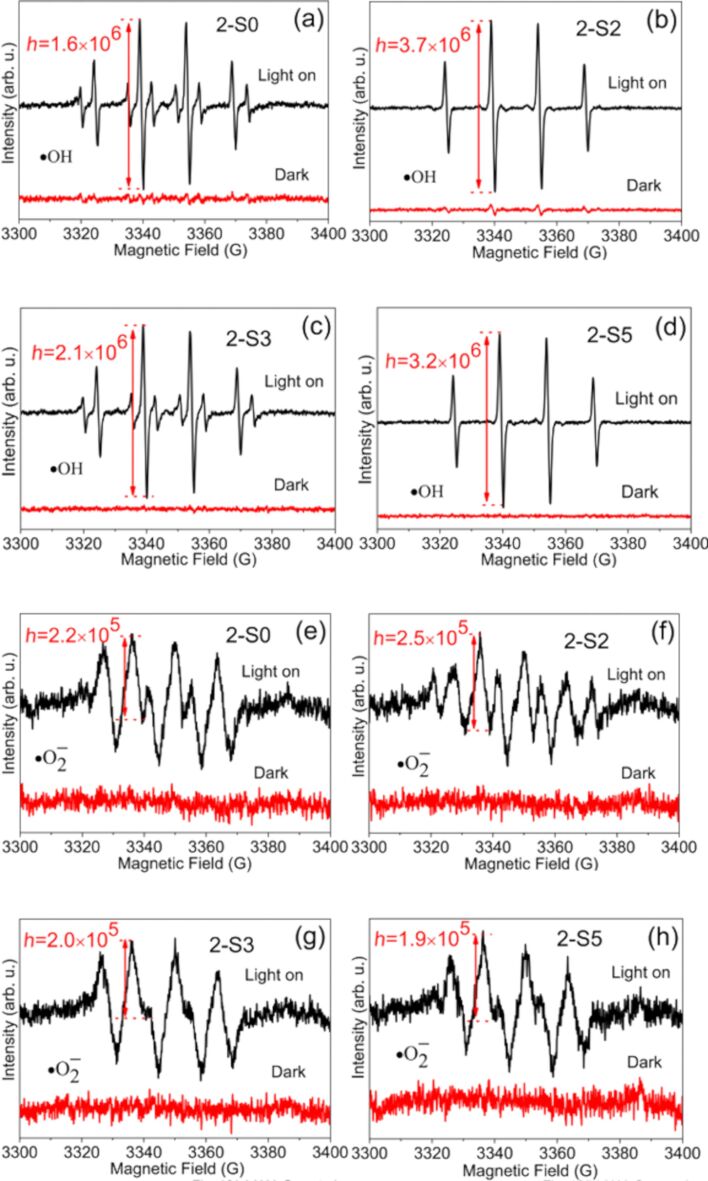
ESR spectra of radical adducts trapped by DMPO in 2-S0 (a, e), 2-S2 (b, f), 2-S3 (c, g), and 2-S2 (d, h) dispersions: (a, b, c, d) DMPO–•OH formed in aqueous dispersions, (e, f, g, h) DMPO–•O_2_^−^ formed in methanol dispersion.

Figure 10 shows the ESR spectra of the samples 2-S0 (a, e), 2-S2 (b, f), 2-S3 (c, g) and 2-S5 (d, h). Prior to irradiation, there is no obvious signal in the ESP spectra. After irradiation, the superoxide radical (•O_2_^−^) and hydroxyl radical (•OH) signals clearly appear for all the samples. TiO_2_ is excited by photons to generate electron–hole pairs, i.e., TiO_2_ + *h*ν = TiO_2_ + e^−^ + h^+^. Superoxide radicals are then formed by the reaction of the electrons with the O_2_ adsorbed at the surface of the TiO_2_ particles, i.e., e^−^ + O_2_ → •O_2_^−^. The holes react with water or hydroxyl groups to form hydroxyl radicals, i.e., h^+^ + H_2_O →•OH + H^+^ [44].

For the superoxide radical (•O_2_^−^), the peak height (*h*) in the ESR spectrum of sample 2-S0 is 2.2 × 10^5^. As *R*_S/Ti_ increases, the peak height varies between 1.9 × 10^5^ and 2.5 × 10^5^. For the hydroxyl radical (•OH), the peak height varies in the range from 1.6 × 10^6^ to 3.7 × 10^6^. The 2-S2 sample has the highest •OH and •O_2_^−^ signal intensity. This indicates that the sample has the highest number of •OH and •O_2_^−^ radicals. Therefore, it shows the best pollutant degradation performance. •OH and •O_2_^−^ radicals can degrade organic pollutants (MB) into nontoxic CO_2_ and water.

Furthermore, Figure 10 shows that the •OH signal is stronger than the •O_2_^−^ signal, indicating that more photo-generated holes than electrons can transfer to the TiO_2_ surface. For S-doped TiO_2_, the outermost electronic orbitals of the S, O and Ti atoms are 3s^2^3p^4^, 2s^2^2p^4^ and 3d^2^4s^2^, respectively. When S^2−^ replaces O^2−^, it is neither a donor nor an acceptor and has no influence on the carrier concentration in TiO_2_. This is because S and O have the same number of outmost electrons. When *R*_S/Ti_ is 2, S^6+^ begins to replace Ti^4+^ in the 2-S2 sample, whereupon the electron concentration becomes higher than the hole concentration, because S has more outer shell electrons than Ti. If the electrons and holes were both transferred to the surface of the TiO_2_ particles, the •O_2_^−^ signal would be stronger than the •OH signal; however, this is not the case, which could be due to trapping of electrons by impurities or defects.

## Conclusion

S-doped (001)-TiO_2_ with different *R*_S/Ti_ were synthesized by thermal chemical vapor deposition at 180 and 250 °C and systematically characterized by XRD, TEM, FTIR, XPS, UV–vis DRS, PL, BET and ESR.

The S-doped sample produced at 180 °C shows little changes in the structure, morphology, chemical state and photocatalytic properties of undoped TiO_2_, indicating that the temperature is not high enough to effectively achieve S-doping. The effects of S-doping at 250 °C are as follows: (1) S-doping induces a crystal lattice distortion, and the ratio of the lattice parameters *c*/*a* varies with the *R*_S/Ti_ ratios, reaching a maximum at *R*_S/Ti_ = 3. This is a result of the different ionic radius of S compared to O and Ti, which are replaced. (2) S-doping changes the morphology of the particles and results in the aggregation of particles; consequently, the specific surface area decreases. (3) S-doping increases the adsorption coefficient *A_e_* from 0.9% to 68.5% due to the increased number of oxygen vacancies and larger amount of SO_4_^2−^ on the surface of the TiO_2_ particles. (4) S-doping increases the degradation rate from 6.9 × 10^−2^ min^−1^ to 18.2 × 10^−2^ min^−1^. This is due to the presence of more •OH and •O^2−^ radicals with high reactivity.
